# The Feebleminded

**Published:** 1920-01-03

**Authors:** Evelyn Fox

**Affiliations:** Hon. Secretary.


### THE FEEBLEMINDED.

*To the Editor of* THE HOSPITAL.

In your issue of December 6 you draw attention to the question of the mentally defective, and express the hope that the problem will soon be tackled effectively.

While in profound agreement with all that is said by you as to the urgency of the problem, I should like to point out that the duty of supervising defectives who can be dealt with under the Mental Deficiency Act is actually laid upon the local authorities. This duty of supervision in their own homes is, however, carried out by our Voluntary Associations, where such exist.

Moreover, our Associations are the only means of helping the friends and guardians of defectives who do not come under the Act, and there is no doubt that many defectives who should have training or education provided from public funds are left entirely to voluntary effort.

The Central Association for the Care of the Mentally Defective (Incorporated) was formed early in 1914 as a result of the passing of the Mental Deficiency Act, and was formed with the purpose of: (1) Aiding local authorities in the administration of the Act; (2) caring for and befriending those defectives who do not come under the Act; (3) training teachers and social workers in the care of defectives; (4) forming associations throughout the country to visit defectives in their own homes.

The Association works in close co-operation with the Government Department concerned—the Board of Control—and receives a yearly grant from them. We, therefore, have the twofold advantage of Government support and recognition with the freedom and elasticity which a voluntary and independent body can enjoy.

During the war the Association had perforce to limit its activities, but the time has now come when the Council feel an expansion of the work is not only advisable, but imperative, and an appeal for financial help towards further development is shortly to be made.

EVELYN FOX, Hon. Secretary.

December 22, 1919.

